# Using minimally invasive aesthetic repair, dental fluorosis vertical distance of occlusion restoration: A case report

**DOI:** 10.1097/MD.0000000000046415

**Published:** 2025-12-12

**Authors:** Jiaxin Hu, Yi Luo

**Affiliations:** a Hospital of Stomatology Shantou University Medical College, Shantou, China; b Guiyang Hospital of Stomatology, Guiyang, China.

**Keywords:** lithium disilicate ceramic, minimally invasive dentistry, occlusal reconstruction, tooth wear

## Abstract

**Rationale:**

While full-coverage crowns are the conventional solution for restoring severely compromised vertical distance of occlusion restoration, this approach is inherently invasive. This report details a conservative alternative using veneers in a dental fluorosis patient – a clinical scenario seldom documented in developed regions.

**Patient concerns:**

A 57-year-old patient from a high-fluoride area presented with impaired masticatory function and severe occlusal wear.

**Diagnoses:**

Severe attrition of the upper and lower dentition. Severe dental fluorosis.

**Interventions:**

A minimally invasive protocol was selected, involving the restoration of the vertical dimension and occlusal function with veneers.

**Outcomes:**

This case demonstrates that minimally invasive veneer rehabilitation can successfully manage severe occlusal wear in dental fluorosis, reestablishing functional occlusion and aesthetics while preserving vital tooth structure. It represents a viable, tooth-preserving alternative to conventional full-coverage crowns.

**Lessons:**

Compared to traditional full-coverage crowns, the presented minimally invasive approach offers the distinct advantage of preserving sound tooth structure, which is a cornerstone of contemporary adhesive dentistry. This is particularly pertinent for fluorotic teeth, where maximizing the retention of healthy enamel is desirable.

## 1. Introduction

Severe tooth wear presents a significant clinical challenge, often leading to reduced vertical dimension of occlusion (VDO), impaired masticatory function, and temporomandibular joint (TMJ) disorders.^[1]^ Additionally, the link between masticatory function and cognitive health suggests that addressing such dental issues may have broader systemic implications.^[2,3]^ While full-coverage crown restoration is the conventional standard for rehabilitating severely worn dentitions, its invasive nature necessitates substantial removal of sound tooth structure. The evolution of adhesive dentistry has paved the way for minimally invasive alternatives, such as veneer restorations, which aim to preserve healthy tissue.^[4]^ However, their application in complex cases like severe dental fluorosis remains less explored.

The management of dental fluorosis poses unique difficulties due to its aberrant enamel structure, which is characterized by a hypermineralized surface layer over a porous, hypomineralized subsurface.^[5,6]^ This compromised substrate challenges the formation of a reliable resin-bonded interface, a cornerstone of minimally invasive dentistry.^[7]^ Furthermore, in cases of severe wear, the critical clinical step of determining a physiologically acceptable and stable jaw relationship VDO is paramount for successful occlusal rehabilitation.^[8,9]^ A reversible diagnostic phase is crucial for this assessment.^[10]^

This report details a successful full-mouth rehabilitation using minimally invasive veneers in a patient with dental fluorosis and severely worn dentition. It highlights the clinical strategy for overcoming bonding challenges and establishing a stable VDO, demonstrating a viable, tooth-preserving protocol for a condition where conventional crowns are typically employed.

## 2. Case presentation

### 2.1. Medical history and chief complaint

A 57-year-old Asian man presented to the Stomatological Hospital of Guiyang, China, with the chief complaint of “ineffective chewing and sensitivity in the posterior teeth for the past two years,” which had significantly compromised his normal diet and quality of life. The patient reported growing up in Bijie, China, a known high-fluoride area.

### 2.2. Clinical examination

#### 2.2.1. Facial and jaw examination

**Figure 1.**
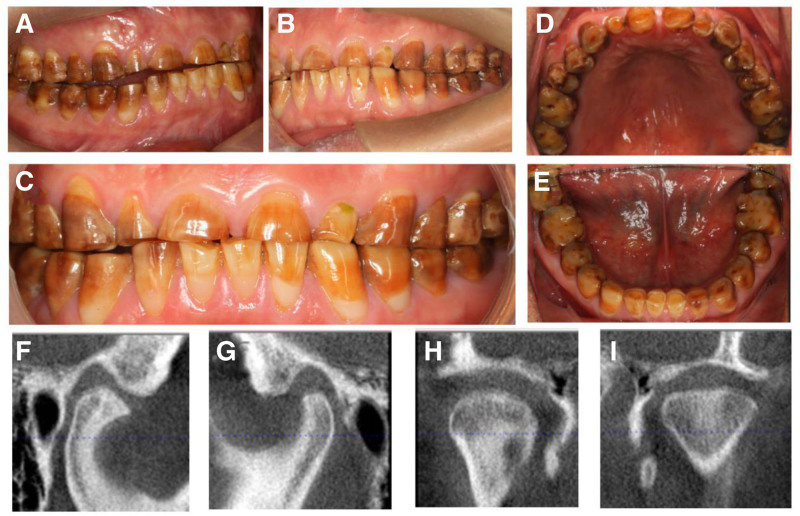
Preoperative examination. (A and B) Preoperative oral side bit: the centric relation position, with no occlusal contact on the right side. (C) Preoperative oral position: interdental dislocation, front teeth with edge-to-edge occlusion. (D and E) Preoperative oral occlusion: large area of dark brown staining and pitting defect in enamel. (F) CBCT coronal position: right temporomandibular coronal position. (G) CBCT coronal position: left TMJ. (H) CBCT sagittal position: left TMJ. (I) CBCT sagittal position: right TMJ. CBCT = cone beam CT, TMJ = temporomandibular joint.

The patient presented with facial asymmetry and a mandibular deviation to the right (Fig. 1A and B). A habitual right lateral shift of the mandible was observed, enabling him to achieve a functional intercuspation; otherwise, occlusal contact on the right side was absent.

The patient had an anterior edge-to-edge occlusion (Fig. 1C), resulting in occlusal instability. A discrepancy was confirmed between the centric relation position and the habitual intercuspation position achieved via the mandibular shift. The lower facial height was measured at 58.21 mm, with a maximum mouth opening of 41 mm.

#### 2.2.2. Oral examination

The dentition exhibited severe attrition. The tooth surfaces displayed obvious yellowish-brown stains and varying degrees of enamel defects, consistent with a diagnosis of dental fluorosis (Fig. 1D and E).

#### 2.2.3. TMJ examination

No tenderness, clicking, or crepitus was detected in the TMJ upon palpation. The masticatory muscles were also non-tender.

### 2.3. Radiographic examination

Cone beam computed tomography imaging revealed no evidence of degenerative changes, motion limitations, or other pathologies in the TMJs. The cortical outlines of the bilateral condyles were continuous and intact (Fig. 1F and I).

### 2.4. Diagnosis

Based on the clinical and radiographic findings, the patient was diagnosed with:

Severe attrition of the upper and lower dentition.
Severe dental fluorosis.

### 2.5. Repair scheme before treatment

The conventional treatment for such extensive wear and occlusal collapse would often involve full-coverage crowns. However, a minimally invasive approach using lithium disilicate veneers was selected based on the following considerations:

#### 2.5.1. Principle of maximum tissue preservation

A primary therapeutic goal was to conserve the remaining, albeit fluorotic, tooth structure. Veneers, requiring only minimal enamel reduction, align with this modern adhesive dentistry principle.

#### 2.5.2. Aesthetic and functional potential of veneers

Contemporary high-strength ceramic materials (e.g., lithium disilicate) allow veneers to be utilized for limited occlusal coverage, capable of restoring the vertical dimension and withstanding functional loads in selected cases.

#### 2.5.3. Validation through diagnostic protocol

The feasibility of this approach was first tested and validated using a diagnostic wax-up and a mockup, which confirmed that the desired aesthetic and functional outcomes – including a stable jaw position and occlusal scheme – could be predictably achieved without resorting to full-coverage preparations.

### 2.6. Design and fabrication of bite plates

**Figure 2.**
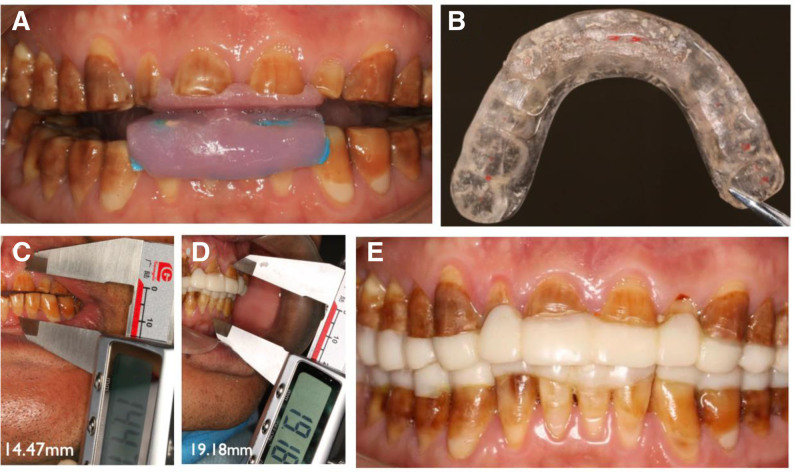
Preparation before final repair. (A) Relaxed bite plate. (B) Stable bite plate. (C) Interarch distance before restoration. (D) Interarch distance after wearing bite plate. (E) The bite plate with dental anatomical shape.

In the oral examination, we found that the patient had a disordered and unstable occlusion. Therefore, the patient was instructed to wear the relaxed bite plate. The purpose of wearing is to relieve the tension of masticatory muscles and guide the centric relation position (Fig. 2A). This kind of bite plate is made of self-setting acrylic resin covering the area of anterior teeth. The plane where the maxillary and mandibular bite plates contact, maintaining uniform contact. On the inner side of the guide plate, light silicone rubber is used to enter the inverted recess to strengthen the retention.

After 2 days of follow-up, it was discovered that the patient had reached a state of masticatory muscle relaxation, and his jaw position relationship could be directed to the centric relation position, allowing the facial arch to be transferred. Then create a stable bite plate with a thickness of around 2 mm (Fig. 2B), allowing it to gradually adjust to the reduced VDO. After wearing it, check for joint and muscle soreness 2 weeks, 1 month, and 3 months later, and make multiple adjustments to get a comfortable occlusal relationship.

At the 3-month follow-up visit, it was confirmed that the patient had achieved a stable centric relation position. At this stage, the bite plate with dental anatomical shape can be manufactured. This helps restore the anatomical form of the teeth and assists the patient in adapting to the new crown morphology and masticatory habits. First, the existing interocclusal distance was measured, 14.47 mm (Fig. 2C), and the vertical dimension to be restored was calculated by considering the minimal restorative space, facial proportions, and the rest position of the mandible. The interocclusal distance needed to be restored to approximately 2 mm. Then, impressions were taken then the dental stone models were produced. The facebow record was transferred to an articulator, and a diagnostic wax-up was fabricated by the dental technician. Based on the diagnostic wax-up, the physician used an indexing technique to fabricate an anatomically contoured occlusal splint intraorally, which was then adjusted to achieve uniform occlusal contacts. Occlusion and interocclusal distance were assessed during the prosthesis try-in and measured at 19.18 mm (Fig. 2D and E). The occlusal scheme of transitional restoration is mutually protected occlusion and canine guidance, which is realized by adjusting occlusion. Throughout the 3-month period, the patient wore the appliance and attended weekly visits with frequent occlusal examinations and adjustments.

After 3 months of ongoing adjustments, the occlusal scheme of the bite plate at the current follow-up visit was deemed most comfortable by the patient. Therefore, the occlusal relationship and contour of this plate were preserved and used to fabricate a new diagnostic wax-up for the subsequent definitive restoration.

### 2.7. Fabrication of the definitive restoration

Following completion of the new diagnostic wax-up, the occlusal splint was removed and the teeth were isolated with a rubber dam. Abutment polishing paste was used to clean the prepared teeth. Teeth preparation of veneer was performed sequentially from the anterior teeth to the premolar and molar regions. Subsequently, impressions were made, the facebow record was transferred, and using an indexing technique derived from the diagnostic wax-up, a provisional resin restoration was fabricated intraorally and provisionally cemented.

Try on all veneers, check fit and seating, and try on veneers with vivolink N clear color reagent after fitting to determine the color of the bonded cement–vivolink N clear matrix cement. The inner surface of the reinforced lithium disilicate glass ceramic IPS e. max press veneers were treated with 9.5% hydrofluoric acid for 80 to 90 seconds, rinsed for 30 seconds, and air-dried to a chalky change in surface color. The Ivoclean was coated on the inner surface of the prosthesis and reacted for 20 seconds, followed by thorough cleaning and drying. Alkylating agent (A&B in 1:1 mixture, Bis-silane Choice2 Starter Kit, BISCO Inc., Schaumburg) was then applied and allowed to dry for 30 seconds.

Using 37% phosphoric acid gel, the abutment was acid etched for 45 to 60 seconds before being cleaned and dried.^[11]^ After the 2 groups have been glued together, be sure there is no gum bleeding. Bonding sequence: Segments of the anterior, premolar, and molar regions were joined one after the other. Each face must be bonded in order for it to be stable, and after bonding, any extra adhesive material must be cleaned up to make sure that none is left behind. Following the bonding of the veneer, an evaluation of the teeth was done. The anterior lateral occlusion was free of interference, the middle occlusion was in uniform contact, and polishing was finished.

### 2.8. Final restorative outcome

**Figure 3.**
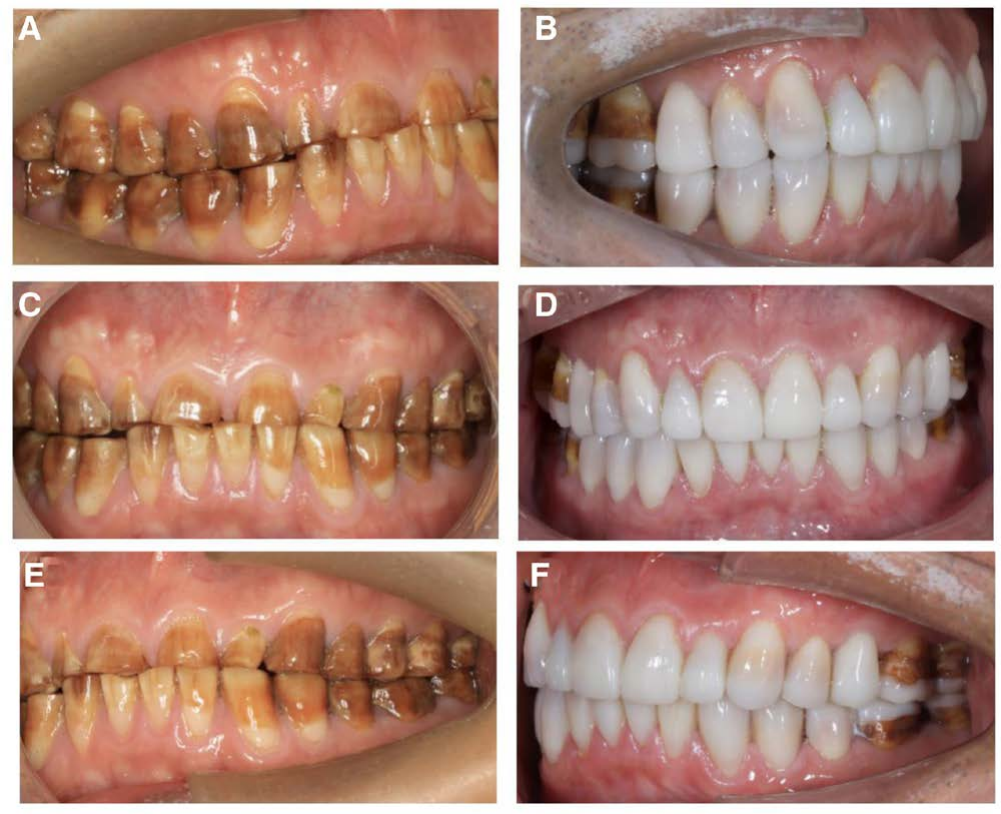
Comparison before and after veneer restoration. (3A vs 3B) Preoperative right lateral view versus postoperative right lateral view. (3C vs 3D) Preoperative frontal view versus postoperative frontal view. (3E vs 3F) Preoperative left lateral view versus postoperative left lateral view.

Both functional and aesthetic outcomes were evaluated at the 2-year follow-up. Aesthetically, the definitive lithium disilicate veneers demonstrated excellent marginal adaptation, seamless color match, and a natural surface texture and characterization, achieving a highly pleasing aesthetic result (Fig. 3). Functionally, the patient reported complete absence of occlusal discomfort or TMJ symptoms. A stable centric relation and mutually protected occlusion were confirmed clinically. Masticatory function was fully restored, allowing the patient to comfortably manage a normal diet.

From the patient’s perspective, the treatment outcome was profoundly positive. He expressed great pleasure with the natural appearance of the restorations and reported a newfound confidence in his smile. Functionally, he highlighted the complete resolution of chewing difficulties, relishing foods he had previously avoided. This restoration of function and aesthetics, in his view, translated directly to a significant uplift in his daily life and well-being.

## 3. Discussion and conclusions

The present case report demonstrates a novel application of minimally invasive veneers for full-mouth occlusal rehabilitation in a patient with severe dental fluorosis and significantly worn dentition. While veneers are well-established for aesthetic corrections,^[12]^ their use to restore the VDO and manage severe wear in fluorotic dentition is scarcely reported, distinguishing the therapeutic strategy described here from conventional crown-based approaches.^[13]^

The management of this case confronted 2 principal challenges. The first was achieving a durable bond to the aberrant enamel structure of fluorotic teeth. The hypermineralized surface layer over a porous, protein-rich subsurface often compromises micromechanical retention. To address this, we employed an extended acid-etching protocol, a technique supported by evidence indicating that prolonged etching can better expose the enamel prism cores in fluorotic enamel, thereby enhancing resin tag formation and improving bond strengths.^[8]^ The second challenge was the accurate determination of a physiological and stable VDO. In cases of extensive wear, this is a critical yet non-trivial step.^[9]^ We utilized a reversible diagnostic protocol involving an occlusal splint and mockup, allowing for clinical validation of the proposed occlusal scheme and jaw position over time before proceeding to definitive treatment.^[11]^ This phased approach mitigates the risk of post-operative discomfort and ensures functional harmony within the masticatory system.

Compared to traditional full-coverage crowns, the presented minimally invasive approach offers the distinct advantage of preserving sound tooth structure, which is a cornerstone of contemporary adhesive dentistry.^[7]^ This is particularly pertinent for fluorotic teeth, where maximizing the retention of healthy enamel is desirable. However, it is crucial to acknowledge the limitations of this report. Firstly, this is a single case, and the long-term performance of veneers under full occlusal load in a significantly raised VDO remains to be fully established through long-term clinical studies and larger cohorts. The existing literature on such applications is limited, making direct comparisons challenging and underscoring the novelty of this case. Secondly, the success of this technique is highly dependent on meticulous case selection, precise clinical execution, and a robust adhesive protocol, rendering it technique-sensitive. It may be less suitable for patients with preexisting pulpitis or those who have undergone root canal treatment on posterior teeth, where the structural integrity of the tooth is further compromised. Finally, a potential strategy to manage treatment-induced sensitivity would be staged laser desensitization before tooth preparation, although this was not implemented in the present case.^[14,15]^

## Acknowledgments

The authors thank the staff of the Guiyang Hospital of Stomatology. Thanks to the financial support of Hospital of Stomatology Shantou University Medical College.

## Author contributions

**Data curation:** Yi Luo.

**Supervision:** Yi Luo.

**Writing – original draft:** Jiaxin Hu.

**Writing – review & editing:** Jiaxin Hu.
